# Fast‐Rate Capable Electrode Material with Higher Energy Density than LiFePO_4_: 4.2V LiVPO_4_F Synthesized by Scalable Single‐Step Solid‐State Reaction

**DOI:** 10.1002/advs.201500366

**Published:** 2015-12-29

**Authors:** Minkyung Kim, Seongsu Lee, Byoungwoo Kang

**Affiliations:** ^1^ Department of Materials Science and EngineeringPohang University of Science and Technology (POSTECH)Pohang790‐784Republic of Korea; ^2^ Korea Atomic Energy Research InstituteP.O. Box 105Yuseong‐guDaejeon305‐600Republic of Korea

**Keywords:** F‐containing compounds, high power material, lithium ion batteries, LiVPO_4_F, PTFE as additional fluorine source

## Abstract

Use of compounds that contain fluorine (F) as electrode materials in lithium ion batteries has been considered, but synthesizing single‐phase samples of these compounds is a difficult task. Here, it is demonstrated that a simple scalable single‐step solid‐state process with additional fluorine source can obtain highly pure LiVPO_4_F. The resulting material with submicron particles achieves very high rate capability ≈100 mAh g^−1^ at 60 C‐rate (1‐min discharge) and even at 200 C‐rate (18 s discharge). It retains superior capacity, ≈120 mAh g^−1^ at 10 C charge/10 C discharge rate (6‐min) for 500 cycles with >95% retention efficiency. Furthermore, LiVPO_4_F shows low polarization even at high rates leading to higher operating potential >3.45 V (≈3.6 V at 60 C‐rate), so it achieves high energy density. It is demonstrated for the first time that highly pure LiVPO_4_F can achieve high power capability comparable to LiFePO_4_ and much higher energy density (≈521 Wh g^−1^ at 20 C‐rate) than LiFePO_4_ even without nanostructured particles. LiVPO_4_F can be a real substitute of LiFePO_4._

## 1 Introduction

Lithium ion batteries (LIBs) are used to power many portable electronic devices and large‐scale systems such as electric vehicles and energy‐storage system (ESS). LIBs for powering electric vehicles and ESS must be safe and have high energy density. To meet these requirements, polyanion compounds^1^ such as LiFePO_4_ phosphates,^2^, ^3^ LiMBO_3_ borates,^4^ Li_2_MSiO_4_ silicates,^5^ and Li_2_Fe(SO_4_)_2_ sulfates^6^ have been developed. These compounds have high structural and thermal stability due to strong covalent bonds with oxygen atoms. However, they suffer from lower energy density than layered oxides compounds because polyanion compounds can have low redox potentials or because they can rely on heavy anions such as P and Si. To increase energy density of polyanion compounds, an introduction of fluorine (F) into compounds was suggested as a way to increase the redox potential by exploiting the inductive effect.^7^ Among the F‐containing compounds, LiVPO_4_F has been attracted because of its high redox potential, ≈4.2V (vs Li^+^/Li^0^), the highest redox potential among V^3+^/V^4+^ redox couples in polyanion compounds.^8^ It achieves ≈655 Wh kg^−1^ of energy density, which is higher than that of LiFePO_4_, 595 Wh kg^−1^.^9^ LiVPO_4_F also has good thermal and structural stability comparable to that of LiFePO_4_ 10 and has been reported as a promising high‐rate cathode material in calculation^11^ but this prediction has not been confirmed experimentally yet.

Nevertheless, LiVPO_4_F has not been studied as much as other polyanion compounds because of difficulties in synthesis_._ To obtain phase‐pure LiVPO_4_F, a carbon thermal reduction (CTR) reaction is typically used along with two‐step heat treatments.^12^, ^13^, ^14^ In the first heat treatment, CTR reaction uses a large amount of carbon (C) to produce VPO_4_/C without oxidation of vanadium, which then reacts with LiF to obtain LiVPO_4_F/C. During the second heat treatment, rapid calcination (hold for 15 min) and rapid cooling (or quenching) are typically applied to prevent formation of impurity phases such as Li_3_V_2_(PO_4_)_3_.^12^, ^15^ The amount of carbon in VPO_4_/C should be carefully measured to obtain the exact mole ratio of elements in LiVPO_4_F because a deviation from stoichiometry easily leads to formation of the impurity such as Li_3_V_2_(PO_4_)_3_.^15^

Furthermore, the quenching process in CTR process can lead to the oxidation of LiVPO_4_F such as the formation of LiVOPO_4_. During quenching, a sample can be exposed to air, which can oxidize particles and lead to formation of LiVPO_4_O on the surface or in the bulk.

The formation of impurity phases such as Li_3_V_2_(PO_4_)_3_ and LiVPO_4_O should be avoided. Many efforts to synthesize LiVPO_4_F directly without CTR reaction and quenching have been attempted. However, most approaches such as simple chemical lithiation along with postannealing,^16^ and solid‐state reaction with mechanical activation^17^ have been focused on avoiding the formation of Li_3_V_2_(PO_4_)_3_ rather than the formation of the oxidized phase on the surface or in the bulk.

Since impurity phases and oxidation of the bulk or surface can easily occur during synthesis of LiVPO_4_F, its electrochemical properties strongly depend on synthesis conditions. For example, LiVPO_4_F obtained using sol–gel process does not show the characteristic electrochemical feature of LiVPO_4_F such as the voltage‐step behavior in charge process even though it does not have any impurity phase such as Li_3_V_2_(PO_4_)_3_ in XRD.^18^ This absence of voltage step may be from the formation of the oxidized phase, LiVOPO_4_ that does not show any voltage step behavior. In addition, the effect of the oxidized phase on electrochemical properties of LiVPO_4_F can be much severe than that of Li_3_V_2_(PO_4_)_3_ because the oxidized phase (LiVOPO_4_) typically shows poor electrochemical activity whereas Li_3_V_2_(PO_4_)_3_ shows good electrochemical property. Therefore, the incorporation of the oxidized phase, LiVOPO_4_, into LiVPO_4_F during synthesis strongly affects electrochemical activity of LiVPO_4_F. Thus, control of the synthesis process without oxidation of bulk or surface is very important requirement for improving electrochemical activity of LiVPO_4_F.

Furthermore, recent theoretical calculation has suggested that LiVPO_4_F can be a fast 1D Li diffuser and achieve high rate capability.^11^ Electrochemical properties in 1D Li diffusion compounds such as LiFePO_4_ strongly depend on the surface characteristics^3^ and the particle size,^19^ which can be controlled by adjusting the synthesis conditions. Considering 1D lithium diffusion feature, the oxidation of LiVPO_4_F, especially in the surface can severely affect electrochemical activity. To achieve the electrochemical properties of LiVPO_4_F as predicted in calculation, surface characteristics such as surface oxidation and the particle size should be controlled.

In this study, we report a scalable single‐step solid‐state process to synthesize high‐purity LiVPO_4_F that does not have the impurity phase such as Li_3_V_2_(PO_4_)_3_ and minimizes the oxidation of LiVPO_4_F and report its excellent electrochemical properties. To obtain highly pure LiVPO_4_F without the impurity phase and the oxidation, we added polytetrafluoroethylene (PTFE) as an additional F source that can compensate the loss of fluorine and can create fluorine‐rich environment throughout synthesis. The resulting material shows superior rate capability on discharge up to 200 C rate (18 s discharge) with high operating potential at high rates and retains ≈120 mAh g^−1^ of capacity at 10 C charge/10 C discharge (6 min) for 500 cycles without a significant decay, and can therefore achieve higher energy density than can LiFePO_4_ nanoparticles.

## 2 Result

### 2.1 Origin of Impurity Phases in Single‐Step Solid‐State Reaction

To obtain phase‐pure LiVPO_4_F using single‐step solid‐state reaction, the mechanisms by which impurity phases such as Li_3_V_2_(PO_4_)_3_ originate must be understood. Li_3_V_2_(PO_4_)_3_ is believed to form due to decomposition of LiVPO_4_F as 3LiVPO_4_F → Li_3_V_2_(PO_4_)_3_ + VF_3_ (gas)^15^ but no evidence has been presented to support this idea. To observe weight change during decomposition of LiVPO_4_F, TGA measurement was performed up to 800 °C on as‐prepared LiVPO_4_F, then the decomposed product was carefully characterized by X‐ray diffraction (XRD) (Figure S1, Supporting Information). Under Ar atmosphere, both V_2_O_3_ and Li_3_V_2_(PO_4_)_3_ were the main decomposition products instead of only Li_3_V_2_(PO_4_)_3_. The presence of V_2_O_3_ indicates that F can be easily evaporated, and that oxygen can be involved in the decomposition during synthesis. Furthermore, weight loss of 13 wt% closely matches the theoretical weight change obtained from V_2_O_3_ formation reaction with Li_3_V_2_(PO_4_)_3_. Both F loss and the reaction of oxygen with the sample can lead to formation of impurity phases in as‐prepared LiVPO_4_F, so to obtain pure LiVPO_4_F this decomposition should be minimized.

To develop a scalable “single‐step” synthesis process, all precursors (NH_4_H_2_PO_4_, LiF and V_2_O_5_) were mixed together and then annealed at 700 °C for 1 h in Ar atmosphere. In this process, LiVPO_4_F was successfully synthesized along with impurity phases Li_3_V_2_(PO_4_)_3_ and V_2_O_3_, which are the same as decomposition products of LiVPO_4_F (Figure S2, Supporting Information).

**Figure 1:**
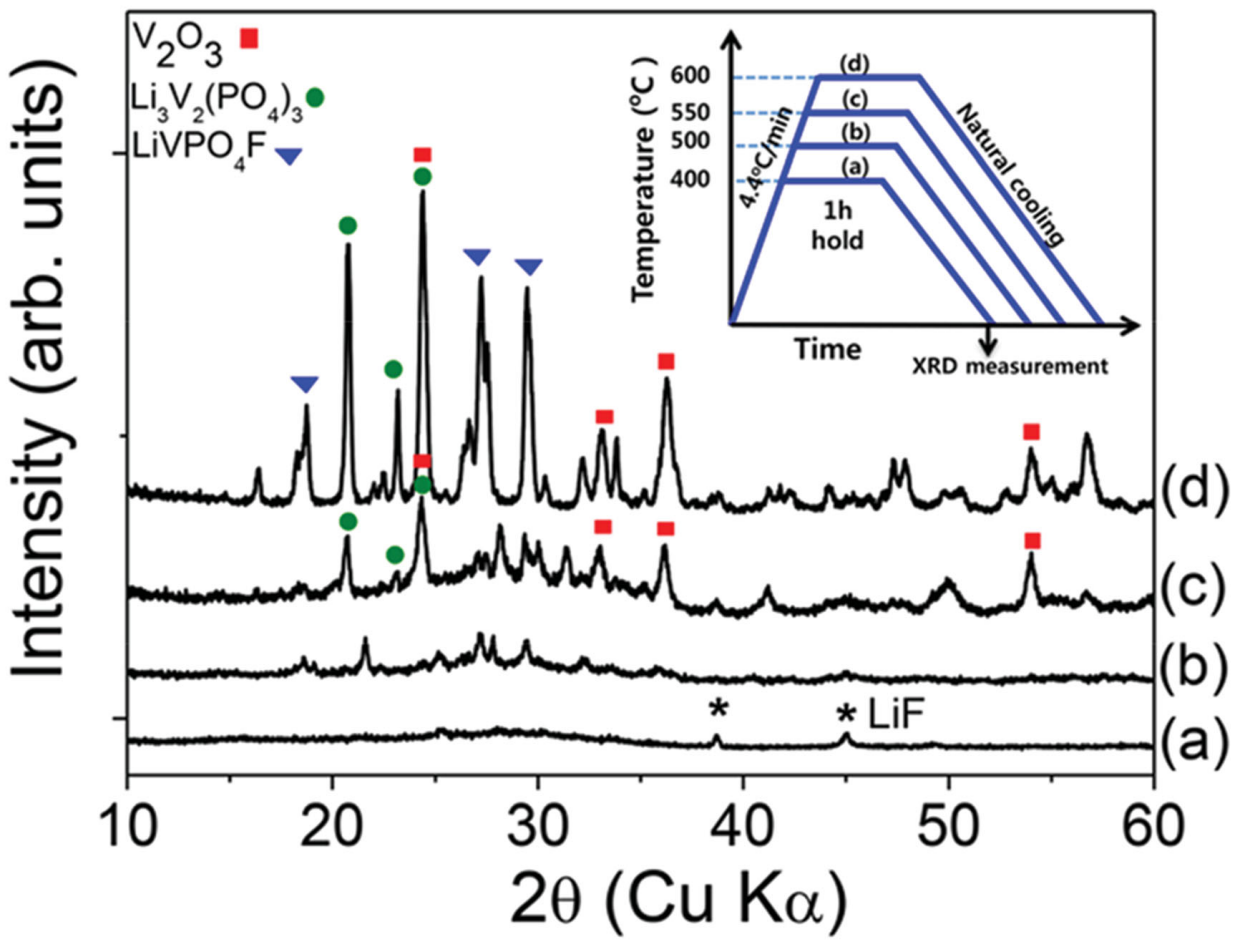
XRD patterns of the samples synthesized for 1 h under Ar at different temperatures (*T*_a_). a) 400 °C, b) 500 °C, c) 550 °C, and d) 600 °C. Inset shows the schematic of annealing process. All precursors were mixed and annealed. Ramping rate was 4.4 °C min^−1^; the samples were held for 1 h at different *T*_a_ (inset). Black star (*) represents LiF peaks.

The reaction pathway was observed at different temperatures to determine whether the impurity phases in a single‐step process were from the decomposition or other reactions during synthesis. A series of experiments was performed under the same conditions but in different annealing temperatures *T*_a_ (Inset of **Figure** **1**). The mix of precursors was annealed in Ar atmosphere for 1 h with the same ramping rate to *T*_a_ = 400 °C, 500 °C, 550 °C, and 600 °C. The samples annealed at different *T*_a_ in the single‐step process were carefully characterized using XRD (Figure 1). When the samples were annealed at 400 °C for 1 h, all precursors except LiF were reacted to form an amorphous phase (hump peak in Figure 1a). As *T*_a_ was increased to 500 °C, LiF completely disappeared and reacted with the amorphous phase to form an unidentified intermediate phases (Figure 1b). When *T*_a_ reached 550 °C, V_2_O_3_ started to appear (Figure 1c). A careful examination of XRD pattern revealed the formation of Li_3_V_2_(PO_4_)_3_ because one of the main peaks of Li_3_V_2_(PO_4_)_3_ overlaps with V_2_O_3_ at 2*θ* = 24.3°. At 600 °C, LiVPO_4_F finally formed and at the same time the amount of Li_3_V_2_(PO_4_)_3_ and V_2_O_3_ was increased (Figure 1d). These results (Figure 1b–d) reveal that the impurity phases, Li_3_V_2_(PO_4_)_3_ and V_2_O_3_, form at relatively lower *T*_a_ than does LiVPO_4_F. The XRD patterns demonstrate that LiVPO_4_F form at *T*_a_ > 550 °C whereas Li_3_V_2_(PO_4_)_3_ and V_2_O_3_ form at *T*_a_ < 550 °C. Since LiF is completely disappeared at 500 °C, the formation of Li_3_V_2_(PO_4_)_3_ and V_2_O_3_ can be related to the loss of F as a result of the decomposition of LiF. As a result, at *T*_a_ < 550 °C, the non‐F compounds such as Li_3_V_2_(PO_4_)_3_ and V_2_O_3_ become more stable than LiVPO_4_F because of their lack of F. In the sample annealed at 600 °C, partial loss of F during the ramping process can lead to formation of the impurity phases. At the same time, residual F can lead to the formation of LiVPO_4_F. Regardless of LiVPO_4_F formation, LiF decomposed at low *T*_a_ and eventually caused the formation of impurity phases.

Thus, to obtain phase pure LiVPO_4_F using the single‐step synthesis process, the loss of F during synthesis, especially at low temperature must be prevented. To do this, additional fluorine (F) should be provided to compensate for the F loss.

### 2.2 Single‐Step Solid‐State Reaction of LiVPO_4_F with PTFE as Additional Fluorine Source

#### 2.2.1 PTFE (Polytetrafluoroethylene ((—CF_2_—CF_2_—)*_n_*) as an Additional Fluorine Source

In this study, PTFE (polytetrafluoroethylene (—CF_2_—CF_2_—)*_n_*) was used as additional F source because when it decomposes at ≈500 °C in inert atmosphere, it can supply only fluorine and carbon.^20^ The decomposition temperature (500 °C–550 °C) matches well with that of LiF decomposition and impurity formation (Figure 1c). Thus, PTFE is suitable to compensate for F loss at 500 °C–550 °C during synthesis. Furthermore, the decomposition of PTFE can create an F‐rich atmosphere throughout synthesis, thereby making LiVPO_4_F more stable than the impurity phases. Carbon in PTFE also can form either CO or CO_2_, thereby retaining an anoxic environment during the synthesis. The amount of impurity formation during the single‐step process strongly depended on the amount of PTFE (Figure S3, Supporting Information). To produce large amount of highly pure LiVPO_4_F, 25 wt% of PTFE was chosen in this study. Additional PTFE (25 wt% of total weight of other precursors in this study) was mixed with other precursors (V_2_O_5_, NH_4_H_2_PO_4,_ and LiF), then the mixture was annealed at 700 °C for 1 h under Ar (3 h ramping to 700 °C). This simple single‐step solid‐state reaction yielded phase‐pure LiVPO_4_F.

#### 2.2.2 Reaction Pathway of Scalable Single‐Step Process with PTFE

**Figure 2:**
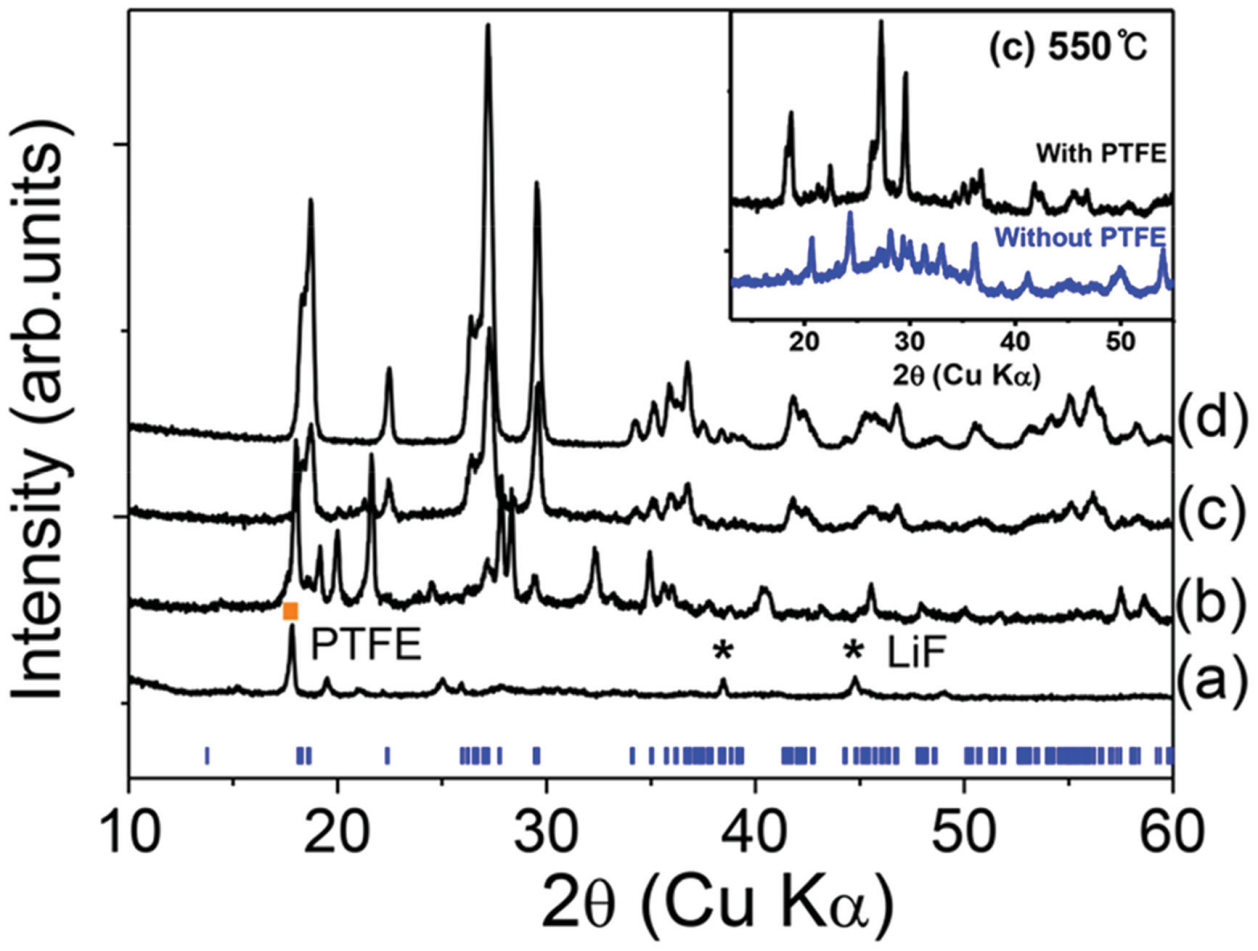
XRD patterns of the samples synthesized with PTFE annealed in the single‐step process at different temperatures (*T*_a_). a) 400 °C, b) 500 °C, c) 550 °C (d) 600 °C for 1 h. Inset: XRD patterns of intermediate phases “with PTFE” and “without PTFE” at 550 °C. Black star (*) represents LiF peaks and yellow square (▪) represents PTFE peak. Blue vertical line is theoretical pattern of LiVPO_4_F (LiVPO_4_F structure information was used from Mba et al.^15^.

Experiments were performed under different *T*_a_ to verify that PTFE can compensate for the F loss especially at 500 °C–550 °C. The mix of precursors and PTFE was annealed in Ar atmosphere for 1 h and ramped up to *T*_a_ = 400 °C, 500 °C, 550 °C, or 600 °C at the same ramping rate. The procedure was the same as the previous one (Inset of Figure 1). XRD patterns of the samples annealed at different *T*_a_ with PTFE (**Figure** **2**) clearly demonstrate that additional PTFE source changed the reaction pathway and then produced phase‐pure LiVPO_4_F.

At 400 °C, some PTFE and LiF remained (Figure 2a); at 500 °C they completely decomposed and formed intermediate phases that were not identified (Figure 2b). At 550 °C, LiVPO_4_F phase started to form instead of V_2_O_3_ and Li_3_V_2_(PO_4_)_3_ (Figure 2c); after PTFE decomposed, impurity phases such as V_2_O_3_ and Li_3_V_2_(PO_4_)_3_ did not form; this result indicates that adding PTFE strongly affects the reaction pathway of LiVPO_4_F. The changed reaction pathway suppresses formation of impurities, and stabilizes LiVPO_4_F at 550 °C. As a result, LiVPO_4_F forms at relatively low temperature (≈550 °C) in the PTFE‐used sample whereas impurity phases formed at around 550 °C in the sample without PTFE (Inset of Figure 2). The F‐rich atmosphere created from the decomposition of PTFE at 550 °C can make F‐containing compounds more stable than non‐F compounds. When *T*_a_ was increased to 600 °C, the peak intensity of LiVPO_4_F produced from the PTFE‐used sample increased greatly; this change indicates that its crystallinity was improved. At 600 °C when PTFE was used, only highly pure LiVPO_4_F formed even without the quenching process. The single‐phase LiVPO_4_F in the presence of PTFE demonstrated that PTFE could help to create an F‐rich environment and thereby compensate for the loss of F. With PTFE, the single‐step process does not require rapid calcination or quenching to obtain pure LiVPO_4_F. Thus, this single‐step synthesis with PTFE can be a practical and scalable method to produce F‐containing compounds and can make LiVPO_4_F promising as a positive material.

#### 2.2.3 Minimization of Surface Oxidation in Single‐Step Solid‐State Process with PTFE

To obtain highly pure LiVPO_4_F, bulk or surface oxidation during synthesis in addition to impurity phase formation should be avoided. Because the decomposition of PTFE can create an F‐rich atmosphere throughout synthesis the single‐step process with PTFE can minimize bulk or surface oxidation. In contrast, LiVPO_4_F synthesized by CTR reaction with two‐step heat treatments can involve surface oxidation or bulk oxidation because the process includes a quenching process in air. The oxidation can lead to formation of LiVPO_4_O‐like structure in the surface or in the bulk that can affect electrochemical property.

**Figure 3:**
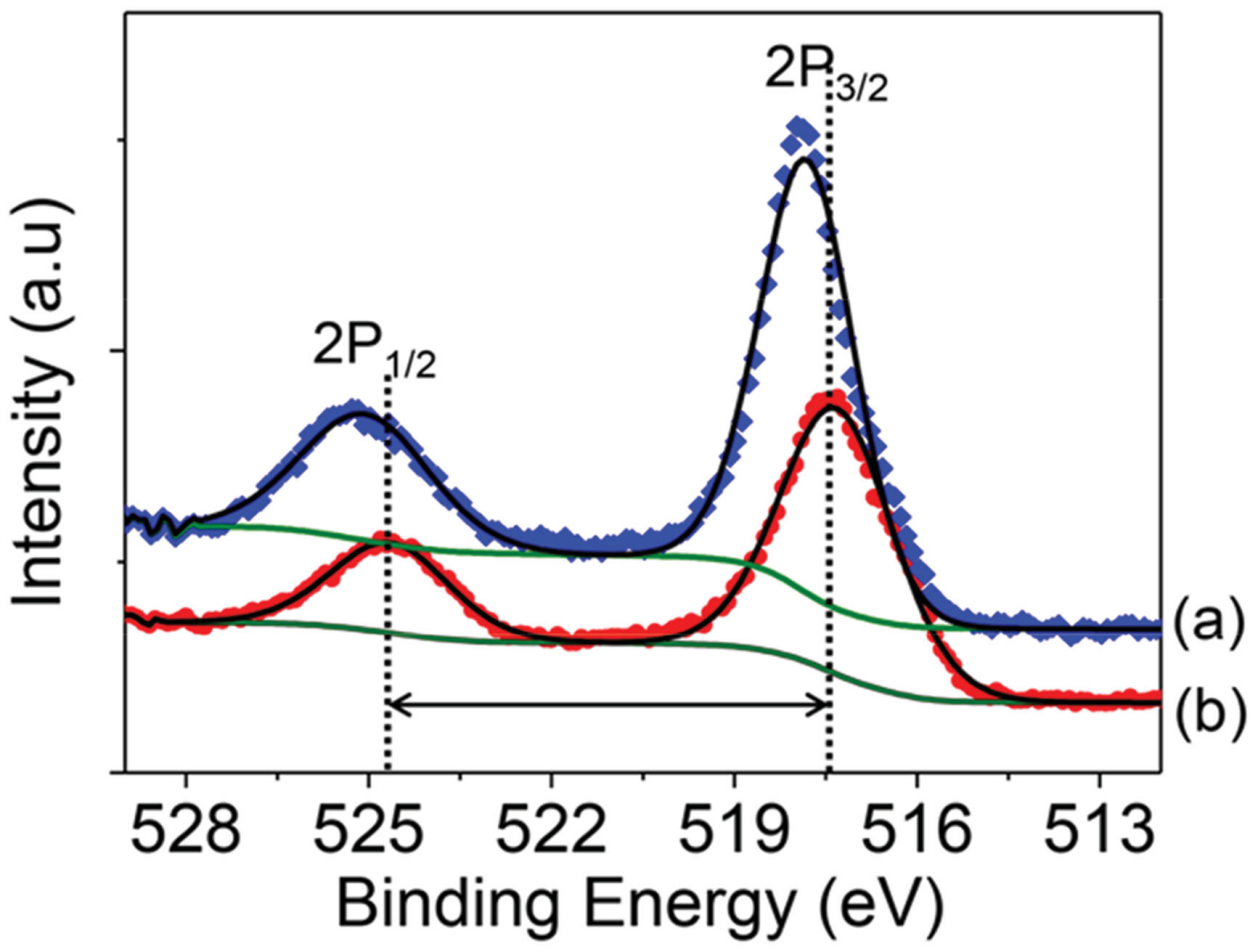
XPS V2p spectra in LiVPO_4_F synthesized by a) CTR process and b) PTFE process. Green lines represent the Shirley‐type backgrounds and thick black lines over the raw data (red circles and blue diamonds) are the summation of the deconvoluted contributions.

To determine whether or not the oxidation depends on the synthesis process, LiVPO_4_F was prepared by CTR reaction with a quenching process in the air (CTR sample). The CTR sample had large amount of carbon (≈12 wt%) because an excess of carbon was added to the precursors to obtain intermediate VPO_4_/C. The surface characteristics of the CTR sample and the PTFE sample were measured by XPS analysis because XRD patterns of the two samples are almost similar with each other (Figure S4, Supporting Information). In V2p spectra in the two samples (**Figure** **3**), the binding energies of the CTR sample were higher than those of the PTFE sample. The binding energies of the CTR sample were 517.80 eV (2p3/2) and 525.05 eV (2p1/2) whereas those of PTFE sample were 517.31 eV (2p3/2) and 524.63 eV (2p1/2) that agree well with published values.^21^ Furthermore, the difference of binding energy between the satellite peak and the main peak was quite different, 7.25 eV for the CTR sample and 7.32 eV for the PTFE sample. The high binding energy of V2p and narrow energy difference of the two peaks (satellite and main peak) in the CTR sample indicate that V has a higher oxidation state than 3+ on the surface. This high oxidation of V indicates that surface oxidation could happen during the quenching process, and lead to formation of a LiV^(IV)^OPO_4_‐like structure on the surface even though the CTR sample had a large amount (≈12 wt%) of carbon. The surface characteristics of LiVPO_4_F strongly depend on the synthesis process even though the two samples did not have Li_3_V_2_(PO_4_)_3_ in the XRD. The developed single‐step process with PTFE can minimize the oxidation, especially surface oxidation without the formation of LiVOPO_4_ on the surface during synthesis because it does not require quenching in the air or rapid cooling and can provide fluorine‐rich atmosphere throughout synthesis.

## 3 Material Characterization

### 3.1 Structural Analysis

**Figure 4:**
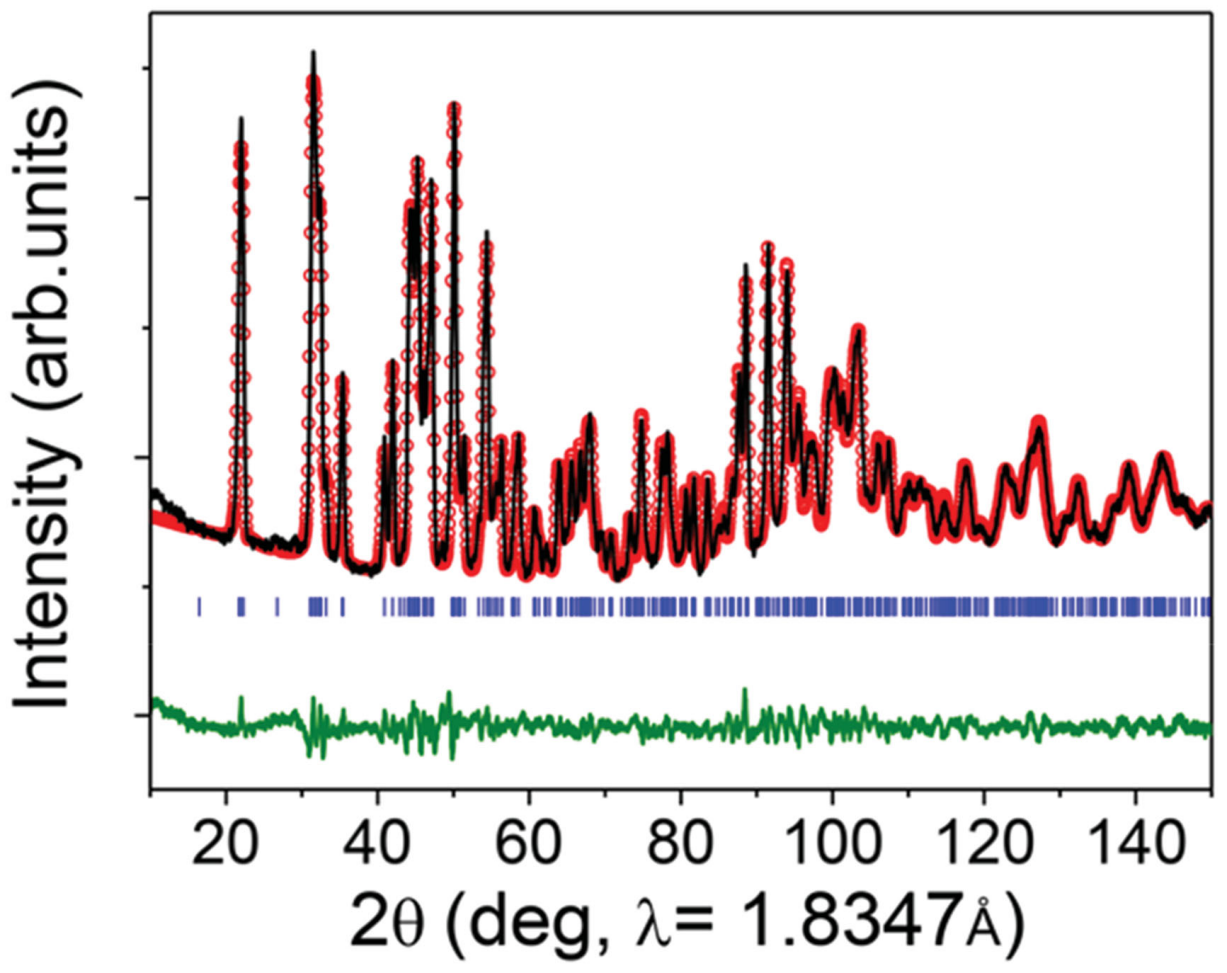
Neutron diffraction pattern of the sample synthesized at 700 °C for 1 h under Ar with PTFE. Observed (red dot), refined (black line), theoretically simulated peak position (blue line) and the difference of observed and calculated value (green line) (*R*_f_ = 1.36, *R*_bragg_ = 2.28, χ^2^ = 5.92).

The crystal structure of the sample synthesized with PTFE was evaluated using neutron diffraction (**Figure** **4**). To perform refinement analysis of neutron diffraction data, two different structures were tried at the starting point; one is taken from LiAlPO_4_F that is isostructure of LiVPO_4_F and have two Li sites^22^ and the other is taken from a recent report that LiVPO_4_F has one Li site.^15^ The structure with one Li site structure was used to refine neutron diffraction data because it led to a better fitting result than did the structures with two Li sites. Lattice parameters of the sample synthesized with PTFE were *a* = 5.1726(4) Å, *b* = 5.3082(3) Å, *c* = 7.2612(5) Å, *α* = 107.5971(6)°, *β* = 107.9643(2)°, *γ* = 98.4061(5)°, and *V* = 174.365 Å3 (Space group: P‐1, *Z* = 2), which are in accordance with the reported values.^15^ Detail structural information of LiVPO_4_F synthesized with PTFE is in Table S1 (Supporting Information).

The bond valence sum was carried out on refinement results of neutron diffraction using the Fullprof program to evaluate the valence states of atoms.^23^ Valence states of atoms are calculated in crystal structure based on the modified second Pauling rule in bond valence sum;^24^ 3.08 for V(1), 3.18 for V(2), 4.77 for P and 0.88 for Li. The valences states of the V atoms were close to ideal value of 3 and smaller than previously reported values.^15^ This result indicates that as‐prepared LiVPO_4_F is close to the ideal structure of LiVPO_4_F.

By using neutron diffraction data, the concentration of antisite defects such as Li/V in two metal sites in the sample synthesized with PTFE was evaluated by using the Rietveld refinement analysis because recent calculation reported that LiVPO_4_F is fast 1D Li diffuser.^11^ Even though V can allow partial occupancy in Li sites or Li can allow partial occupancy in V sites, the refinement result was not further improved. This result can indicate that the concentration of antisite defects in LiVPO_4_F synthesized with PTFE was negligible, possibly because of the single‐step solid‐state reaction that does not include rapid annealing or quenching.

### 3.2 Morphology and Chemical Composition Analysis in the Sample Synthesized with PTFE

**Figure 5:**
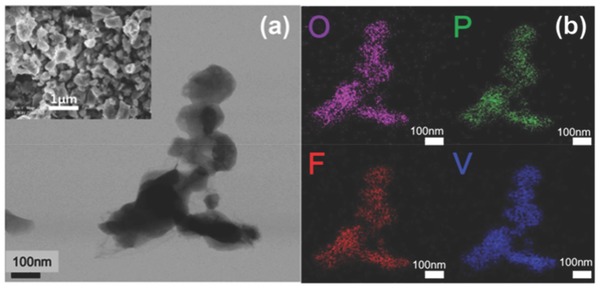
a) TEM image of as‐prepared LiVPO_4_F that was synthesized by a single‐step process with PTFE. Inset is SEM image of LiVPO_4_F. b) EELS image for elemental mapping.

Chemical composition of the sample synthesized with PTFE was measured using ICP‐AES (inductively coupled plasma‐atomic emission spectroscopy). The atomic ratio of V:P:Li was 1.1:1:1.06, which is quite close to the ideal composition ratio. For F analysis, energy‐dispersive X‐ray spectroscopy (EDX) was used. The average value of the elements was V:P:F = 1.07:1:1. Although PTFE could provide excess amount of F, the ratio of F to other atoms was close to 1; this observation indicates that the reaction between PTFE and precursors does not affect the chemical composition of final product. Moreover, the sample synthesized with PTFE contained 1 wt% residual carbon that could be provided when the PTFE is decomposed. In TEM image, the primary particles had faceted shapes and ranged in size from 100 to 200 nm (**Figure** **5**a). Because the single‐step process was completed within 1 h, the size of particles could be restricted to submicron size. Along with the synthesis process, the residual carbon also could inhibit grain growth.^25^ However, the average particle size was 250 nm (range 100–600 nm), which is larger than the primary particle size (inset of Figure 5a); this observation indicates that some particles were agglomerated. In elemental maps by using electron energy loss spectroscopy (EELS) maps, the V, P, O, and F were homogeneously distributed in the bulk of particles (Figure 5b).

## 4 Electrochemical Properties of the Sample Synthesized with PTFE

Electrochemical properties of the sample synthesized with PTFE were evaluated under galvanostatic conditions. LiVPO_4_F synthesized with PTFE (bare‐LiVPO_4_F) shows good rate capability (Figure S7, Supporting Information). Even though bare‐LiVPO_4_F had submicron‐sized particles (100–600 nm) and did not have a large amount of residual carbon (1 wt%), it achieved high rate capability even at 60 C (1 min) discharge rate. The best rate capability of LiVPO_4_F/C reported to date is 10 C (1560 mA g^−1^) rate on discharge with a large amount of carbon in the materials.^9^, ^26^ To further improve electrochemical properties, a carbon coating was utilized with 5 wt% stearic acid by using scalable single‐step solid‐state reaction. The carbon source, stearic acid, was ball‐milled with the other precursors; the rest of process was the same as used to synthesize bare‐LiVPO_4_F. C‐coated LiVPO_4_F had smaller particle size (50–200 nm) and narrower size distribution than did bare‐LiVPO_4_F (Figure S5, Supporting Information). Carbon‐coating layer on the surface of C‐coated LiVPO_4_F particles formed through the process and was amorphous partly due to a low synthesis temperature (Figure S6, Supporting Information). The amount of carbon in the C‐coated LiVPO_4_F sample was ≈5 wt%. Carbon in the sample could further suppress grain growth of particles and thereby lead to submicron particle size.

**Figure 6:**
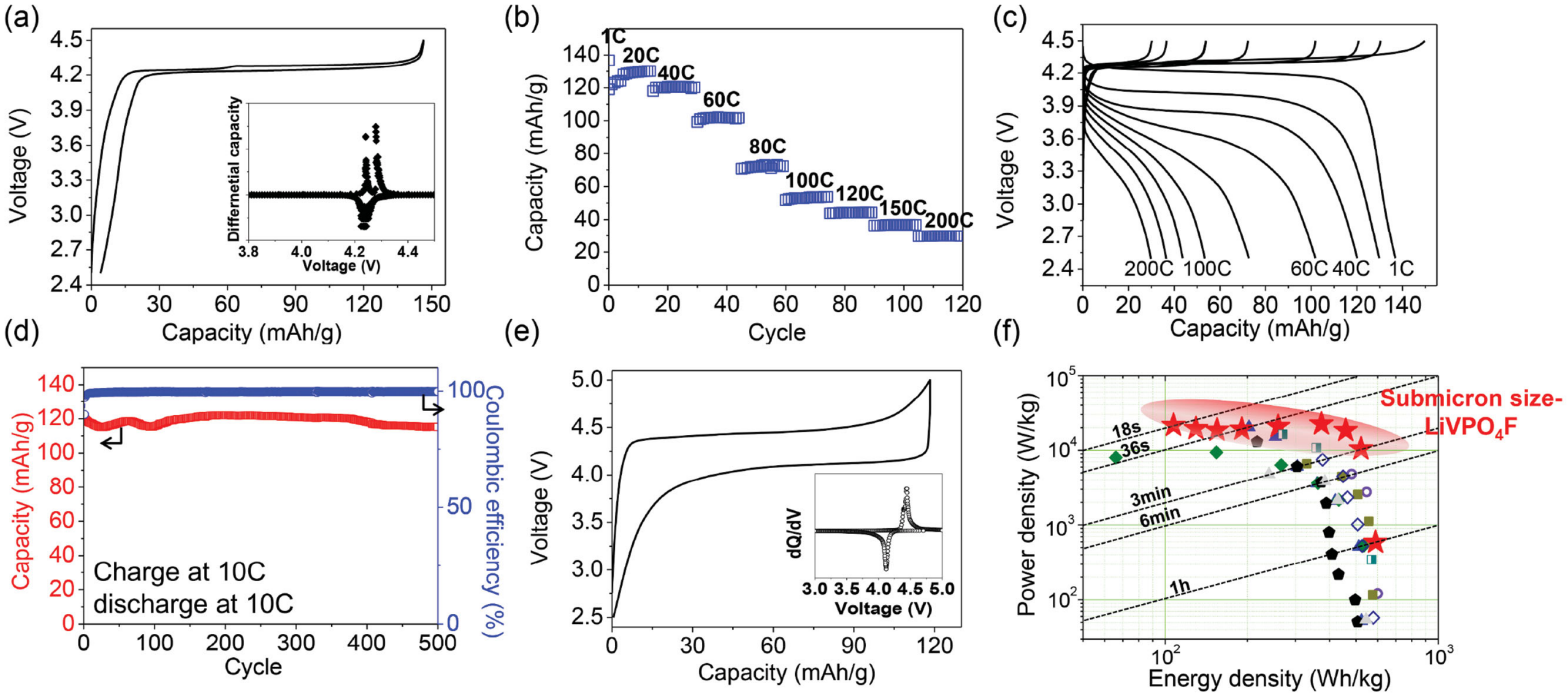
Electrochemical properties of C‐ coated LiVPO_4_F synthesized with PTFE at 700 °C for 1 h under Ar. a) Voltage curve at C/10 rate. (Inset; differential capacity (d*Q*/d*V*) plot of the voltage profile) b) Rate capability at various discharge rates of C‐coated LiVPO_4_F. Cutoff voltage was 2.5–4.5 V. c) Charge and discharge voltage profiles of C‐coated LiVPO_4_F at various rates (right to left: discharging at 1 C, 20 C, 40 C, 60 C, 80 C, 100 C, 120 C, 150 C, and 200 C and charging at 1 C without a voltage hold). d) Capacity retentions at 10 C charge/10 C discharge for 500 cycles. The cutoff voltage was 2.5–5 V (only for cycle retention). e) Voltage profile of 10 C/10 C cycle retention test. (Inset; differential capacity (d*Q*/d*V*) plot of the voltage profile). f) Ragone plot of C‐coated LiVPO_4_F (red stars) in comparison with recent reported nanosize‐LiFePO_4_ and reported LiVPO_4_F data. Green diamond: ref., 30 blue triangle: ref., 39 black pentagon: ref., 40 dark yellow‐square: ref., 41 empty violet circle: ref., 26 half empty green‐square: ref., 42 gray triangle: ref. 43 and empty royal blue diamond: ref. 44 Loading density of the electrode was 2.38–2.6 mg cm^−2^.

A typical voltage curve (**Figure** **6**a) of the C‐coated LiVPO_4_F synthesized with PTFE indicates a V^3+^/V^4+^ redox couple at ≈4.25 V (vs. Li^+^/Li^0^).^27^ During discharge process, the redox potential was 4.23 V. During charge process, the voltage profile of LiVPO_4_F exhibited two steps of potential, at 4.24 V and 4.27 V. This step potential behavior can originate from an intermediate phase, Li_0.67_VPO_4_F,^28^ and indicates that C‐coated LiVPO_4_F undergoes a phase transformation similar to that observed in LiVPO_4_F prepared by CTR reaction with quenching in a sealed gold ampoule.^15^ At C/10 rate, LiVPO_4_F achieved ≈142 mAh g^−1^ at discharge, which corresponds to 91% of theoretical capacity (156 mAh g^−1^).

After reducing particle size and performing carbon coating in LiVPO_4_F synthesis process, the rate capability was substantially improved (Figure 6b). The carbon‐coating process enables LiVPO_4_F to discharge even at 200 C rate (31.2 A g^−1^, 18 s discharge). In a rate capability test, the carbon‐coated LiVPO_4_F was charged at 1 C rate without a voltage hold, i.e., the same as used in the test of bare‐LiVPO_4_F (Figure S7, Supporting Information). The C‐coated LiVPO_4_F shows superior rate capability. Achieved capacity was 136 mAh g^−1^ at 1 C rate, 129.8 mAh g^−1^ at 20 C, 120 mAh g^−1^ at 40 C, and 101.8 mAh g^−1^ at 60 C rate. The superior rate capability is comparable to that of LiFePO_4_. Moreover, at much higher discharging rates, the C‐coated LiVPO_4_F shows reasonable electrochemical activity: 72.7 mAh g^−1^ at 80 C, 53.4 mAh g^−1^ at 100 C, 43.7 mAh g^−1^ at 120 C, 36.4 mAh g^−1^ at 150 C and 29.9 mAh g^−1^ at 200 C rate. Furthermore, regardless of the increased current, the polarization did not substantially increase at high discharge rates. The starting potential was still 3.75 V even at 200 C discharge rate. As a result, C‐coated LiVPO_4_F has higher working potential at high rates (ex. ≈3.6 V at 60 C rate) than does LiFePO_4_ (3.45 V) (Figure S8, Supporting Information), so LiVPO_4_F can have higher energy density than LiFePO_4_ at high rates.

One more interesting point is that particles in LiVPO_4_F of submicron size (50–200 nm) could achieve 200 C discharge rate (18 s discharge); this is in contrast to LiFePO_4_, in which this rate capability can only be obtained by nanosized particles with large amount of carbon in the electrode.^3^ LiVPO_4_F can further improve electrochemical properties in downsizing particle size further.

C‐coated LiVPO_4_F also can achieve high charge rate capability with high capacity. For evaluating fast charge rate capability, a voltage window during cell test was extended from 4.5 to 5.0 V because of 4.2 V redox potential. This can allow LiVPO_4_F to have enough voltage difference in charging process. Under this condition, C‐coated LiVPO_4_F at 10 C charge rate and 10 C discharge rate shows stable capacity retention for 500 cycles (Figure 6d). The capacity was ≈120 mAh g^−1^ at the beginning, then finished to 115 mAh g^−1^ at the 500th cycle; i.e., it retained 95% capacity after 500 cycles. The columbic efficiency was almost 100% for 500 cycles. This prolonged cycle capacity retention indicates that C‐coated LiVPO_4_F has very good structural stability. Furthermore, XRD pattern and SEM of the electrode after 500 cycles clearly show that LiVPO_4_F did not show any structural change and any morphological damage during long cycling (Figure S9, Supporting Information). Also, small polarization was observed in voltage profile of the cycle even at 10 C charge and 10 C discharge rate (Figure 6e). The operating potentials from the differential capacity plot (inset in Figure 6e) were 4.11 V for discharge and 4.44 V for charge. Because of the high operating potential at high rates, LiVPO_4_F achieved both higher power density and energy density than recently reported high power LiFePO_4_ composites and reported LiVPO_4_F compounds (Figure 6e). Surprisingly, power density of C‐coated LiVPO_4_F can be much better than that of nanostructured LiFePO_4_ with conducting agents even though it has submicron particle size without any nanostructure.

In this study, we clearly demonstrate that LiVPO_4_F is a very promising high‐rate capable cathode material with high operating potential and can achieve higher both energy density and power density than those of LiFePO_4._

## 5 Discussion: High Rate Capability of LiVPO_4_F: Smaller Particle Size, Negligible Number of Antisite Defects, and Minimal Surface Oxidation

Use of PTFE as an additional F source during the single‐step solid‐state reaction suppressed formation of impurity phases and minimized surface oxidation, thereby leading to high purity of LiVPO_4_F. C‐coated LiVPO_4_F with submicron particle size can achieve extraordinarily high rate capability up to 200 C rate (18 s discharge) although it has never been reported to have rate capability greater than 10 C discharge rate in literature. To achieve this very high rate capability, LiVPO_4_F does not need nanostructured particles with conducting agents, as does LiFePO_4_. Furthermore, LiVPO_4_F can have higher operating potential at high rates than 3.45 V, redox potential of LiFePO_4_. This outstanding result clearly demonstrates that LiVPO_4_F can be a practical substitute for LiFePO_4_ as a high‐rate capable cathode material and can achieve high energy density at high rates due to high operating potential.^29^, ^30^, ^31^ At 20 C discharge rate, C‐coated LiVPO_4_F can achieve ≈521 Wh kg^−1^ whereas LiFePO_4_ nanostructured particles incorporated with graphene can achieve ≈314 Wh kg^−1^.^29^

LiVPO_4_F has been considered a poor electronic conductor.^26^ However, bare‐LiVPO_4_F with 1 wt% carbon shows good rate capability (even at 60 C rate) than typical LiVPO_4_F/C obtained from previous approaches such as CTR reaction with quenching even though bare‐LiVPO_4_F synthesized PTFE had much lower electronic conductivity (≈2 × 10^−5^ S cm^−1^) than does reported LiVPO_4_F/C (≈10^−2^ S cm^−1^)^26^ by three orders. This finding clearly indicates that the electronic conductivity may not be a critical limiting factor to improve kinetic properties like LiFePO_4_ that high electronic conductivity does not ensure high rate capability.^32^ Like LiFePO_4_, high rate capability in LiVPO_4_F may be a result of fast 1D Li diffusion. Although LiVPO_4_F structurally has a 3D framework, recent calculation^11^ clearly shows that Li ions in LiVPO_4_F diffuse in only one direction with very small activation energy (≈328 meV) that is comparable with the activation energy of LiFePO_4._ 11 Thus, the high rate capability of LiVPO_4_F may be a result of fast 1D Li diffusion. In 1D Li diffusion compounds such as LiFePO_4_, electrochemical properties strongly depend on the particle size, the concentration of antisite defects in two metal sites, and surface characteristics.^3^, ^32^, ^33^ Taking fast 1D Li diffusion characteristic into account, the main cause of substantially improved rate capability in C‐coated LiVPO_4_F could be submicron particle size that is smaller than that of bare LiVPO_4_F and other reported literature values.^9^, ^13^, ^15^ Furthermore, refinement of neutron diffraction data illustrates that the sample synthesized with PTFE has a negligible concentration of antisite defects. A negligible number of antisite defects can improve high rate capability, especially in submicron particles, because the effect of antisite defects on the Li diffusion strongly depends on the particle size in 1D diffusion compounds such as LiFePO_4._ 19 The characteristics of the synthesis process may partly explain why the number of antisite defects in the sample is negligible. The single‐step solid‐state reaction can provide enough energy to rearrange atoms because it does not need rapid sintering that cannot easily provide enough thermal energy, or rapid cooling that does not have enough time for the atoms to diffuse. Recent studies show that the quenched samples in LiVPO_4_F can have more antisite defects than other samples.^34^ Furthermore, LiFePO_4_ can have large amount of antisite defects after low‐temperature synthesis such as hydrothermal or co‐precipitation processes^33^, ^35^ because of low driving force in those processes. In LiVPO_4_F, submicron particle size and the negligible number of antisite defects caused by the developed process can reveal electrochemical properties of LiVPO_4_F as a fast 1D Li diffuser, that lead to high rate capability.

Based on XPS data (Figure 4), the single‐step process with PTFE minimizes surface oxidation that can easily happen during the CTR process even with large amount of residual carbon in the sample and can lead to formation of LiVOPO_4_‐like structures on the surface. In 1D diffusion compounds, surface characteristics of particles strongly limit their rate capability.^3^, ^36^ Formation of LiVOPO_4_ on the surface can severely affect electrochemical activity of LiVPO_4_F with 1D Li diffusion because of the negligible electrochemical activity of LiVOPO_4_.^37^ As a consequence, the minimization of surface oxidation in LiVPO_4_F particles synthesized with PTFE can substantially improve electrochemical activity, and thereby lead to very high rate capability. Considering that C‐coated LiVPO_4_F sample has submicron particles of 50–200 nm size and much smaller surface area, ≈14 m^2^ g^−1^ (BET measurement) than LiFePO_4_ nanopaticles, >30 m^2^ g^−1^,^3^, ^38^ the effect of surface area on exceptional high rate capability can be limited but the characteristic of surface can be more important. By exploiting the high purity of LiVPO_4_F synthesized with PTFE, we have revealed that LiVPO_4_F can achieve superior rate capability as predicted by theoretical calculation. For the first time, we demonstrate that LiVPO_4_F can be a positive material with very high rate capability comparable to that of LiFePO_4,_ and can achieve higher energy density than LiFePO_4_ due to high operating voltage at high rates.

## 6 Conclusions

In this study, additional fluorine source such as PTFE can help to create F‐rich atmosphere throughout synthesis. As a result, highly pure LiVPO_4_F was synthesized with respect to the impurity (Li_3_V_2_(PO_4_)_3_) formation and the oxidized phase (LiVOPO_4_) formation. The single‐step solid‐state synthesis can make synthesis of LiVPO_4_F practical and scalable and thereby establish it as a promising positive material. When synthesized with PTFE, LiVPO_4_F achieved very high rate capability even at 200 C rate (18 s discharge) and stable capacity retention at fast 10 C charge and 10 C discharge rate for 500 cycles even when particles were of submicron size. Furthermore, high operating potential in LiVPO_4_F leads to higher energy density than LiFePO_4_ at high discharge rates. By using scalable single‐step solid‐state process, we clearly demonstrate for the first time that LiVPO_4_F is very fast rate capable material comparable to LiFePO_4_ and can be an electrode material with higher energy density (≈521 Wh kg^−1^ at 20 C rate) than LiFePO_4_ even without nanostructured nanoparticles. We also believe that the scalable single‐step process described here can be further used to obtain other promising F‐containing compounds.

## 7 Experimental Section

*Neutron Diffraction*: Neutron diffraction pattern of LiVPO_4_F were collected at the Korea Atomic Energy Research Institute. The wavelength was 1.8347 Å and the scan range was 10° to 150° in increments of 0.05. The data were collected at RT.

*X‐Ray Diffraction*: X‐ray diffraction was measured to characterize the structure of samples. Two‐step scan powder XRD analysis was performed using a RIGAKU D/MAX‐2500/PC equipped with Cu Kα radiation. Data were collected angles of 10° to 60° at 40 kV and 100 mA, with a step width of 1° or 2° per minute (depending on the sample).

*X‐Ray Photoelectron Spectroscopy*: Surface chemistry of a sample was probed using an XPS spectrometer (ESCALAB250, VG scientific) with Al Kα (1486.8 eV) in a pumped vacuum chamber with a measurement pressure <10^−9^ Torr. CasaXPS (Casa software Ltd) software was used to process the data. The peak in the C 1s region corresponding to adventitious C contamination was set to 285 eV to calibrate binding energy.

*Thermogravimetric Analysis*: Thermal stability of LiVPO4F was analyzed using a TGA/DSC thermogravimeter (SDT Q600 V20.9 Build 20) under Ar atmosphere at a heating rate of 5 K min^−1^ from RT to 800 °C then held for 1 h at 800 °C.

*Scanning Electron Microscope*: A field‐emission scanning electron microscope (FE‐SEM, Philips electron optics B.V, XL30S FEG) was used to characterize the size and morphology of particles in LiVPO_4_F. EDX was used to detect the amount of fluorine of synthesized LiVPO_4_F.

*Transmission Electron Microscopy*: Size of single particles and elemental mapping were achieved using a JEOL JEM‐2100F (Cs Corrector on STEM).

*Inductively Coupled Plasma‐Atomic Emission Spectroscopy*: To determine chemical composition, ICP‐AES (Spectro ARCOS EOP) was used.

*Electrochemical Test*: Swagelok cells were used for electrochemical cell tests. For positive electrode fabrication, the ratio of the composite was active material (LiVPO_4_F): carbon black (Super P): binder (PVDF) = 70:25:5 (wt%). Active material and carbon were handmixed in a mortar for 10 min, then binder and the mixture were stirred together in NMP(*N*‐methyl‐2‐pyrrolidone) solvent for 2 h. The blended slurry was cast using a scalpel onto an aluminum current collector. Then the electrode was dried at 120 °C for 12 h in vacuum. The cathode was cut into a film disk of 8‐mm diameter and approximately 2.38– 2.6 mg cm^−2^ of electrode loading density. The half‐cells were assembled in a glovebox in Ar atmosphere. Li metal was used as the counter electrode and the reference electrode and a microporous polypropylene film (Celgard 2400) was used as a separator. Then 1 m LiPF6 dissolved in mixture of 1:1 ethyl carbonate (EC) and diethyl carbonate (DEC) was used as electrolyte. The electrochemical test was performed with Maccor at RT.

## Supporting information

As a service to our authors and readers, this journal provides supporting information supplied by the authors. Such materials are peer reviewed and may be re‐organized for online delivery, but are not copy‐edited or typeset. Technical support issues arising from supporting information (other than missing files) should be addressed to the authors.

Supplementary
