# Supplementary material for: Fast‐Rate Capable Electrode Material with Higher Energy Density than LiFePO4: 4.2V LiVPO4F Synthesized by Scalable Single‐Step Solid‐State Reaction
Source: Adv Sci (Weinh). 2015 Dec 29;3(3):1500366. doi: 10.1002/advs.201500366 (PMC5064735; doi:10.1002/advs.201500366)
Supplement: Supplementary file 1 — Supplementary [file ADVS-3-1500366-s001.pdf]

Copyright WILEY-VCH Verlag GmbH & Co. KGaA, 69469 Weinheim, Germany, 2015.

ADVANCED SCIENCE
Open Access

Supporting Information

for *Adv. Sci.*, DOI: 10.1002/advs. 201500366

Fast-Rate Capable Electrode Material with Higher Energy Density than $LiFePO_4$: 4.2V $LiVPO_4$ F Synthesized by Scalable Single-Step Solid-State Reaction

*Minkyung Kim, Seongsu Lee, and Byoungwoo Kang**

Supporting Information

Fast rate capable electrode material with higher energy density than $LiFePO_4$: 4.2V $LiVPO_4F$ synthesized by scalable single-step solid-state reaction

*Minkyung Kim, Seongsu Lee and Byoungwoo Kang**

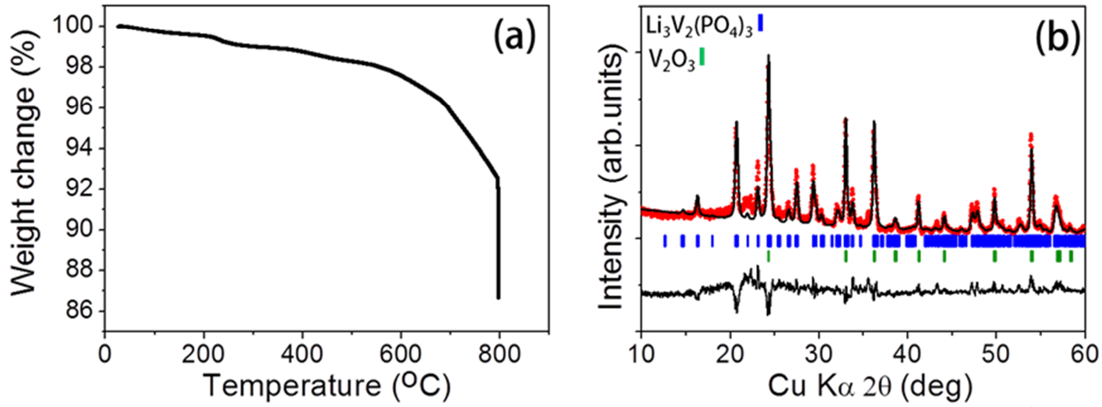

Figure S1. (a) TGA analysis of as-prepared $LiVPO_4F$ up to 800 °C at 5 $Kmin^{-1}$ heating rate and hold at 800 °C for one hour under argon atmosphere (b) XRD pattern of the decomposed product after TGA test.

$$3LiVPO_4F + 1.5O_2 \rightarrow Li_3V_2(PO_4)_3 + 0.5V_2O_3 + 1.5F_2 (\uparrow) \text{ --- } (1)$$

The weight change of $LiVPO_4F$ in TGA was ~ 13wt% that was similar with that of the reaction (1).

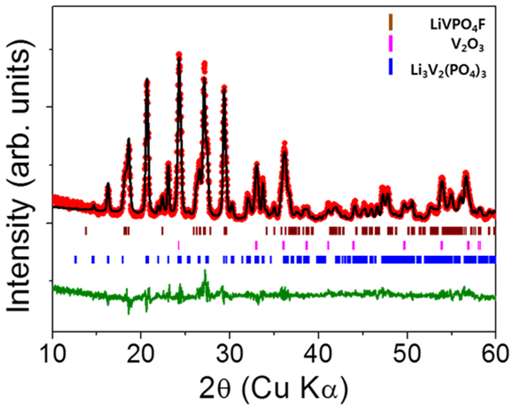

Figure S2. XRD pattern of the sample synthesized by the single-step solid-state reaction at 700 °C with the mixture of precursors (LiF, V_2O_5, and $NH_4H_2PO_4$). The resulting sample was composed of $LiVPO_4F$ and impurity phases, $Li_3V_2(PO_4)_3$ and V_2O_3.

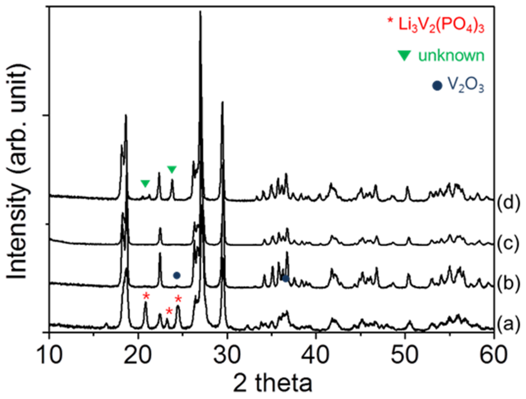

Figure S3. X-ray diffraction patterns of the samples synthesized with different amount of PTFE in the mix of precursors (a) 5 wt% (b) 15 wt% (c) 25 wt% (d) 100 wt%. (700 °C for 1 h under argon)

Phase purity of $LiVPO_4F$ strongly depends on the amount of PTFE in the sample. 5 and 15 wt% of PTFE in the samples was not enough to suppress formation of impurity phases and 100 wt% of PTFE was too much to obtain phase pure $LiVPO_4F$ leading to unidentified impurity phases that can be byproducts of PTFE decomposition.

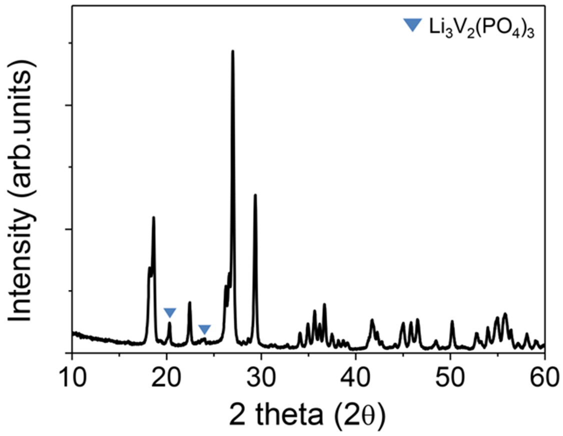

Figure S4. X-ray diffraction patterns of $LiVPO_4F$ synthesized by CTR process. $Li_3V_2(PO_4)_3$ impurity exists.

Table S1. Structural parameters obtained from Rietveld refinement of neutron diffraction pattern from $LiVPO_4F$ synthesized by single-step process with PTFE (25 wt%). (700 °C for 1 h under argon)

**Space group: P-1, Z=2**<br>a = 5.1726 (4) Å, b = 5.3082 (3) Å, c = 7.2612 (5) Å<br>α = 107.5971 (6) °, β = 107.9643 (2) °, γ = 98.4061 (5) °<br>V= 174.365 Å^3			R_f= 1.36<br>R_{braag}=2.28<br>Chi^2= 5.92
	x	y	z
V1	0.00000(0)	0.00000(0)	0.00000(0)
V2	0.00000(0)	0.00000(0)	0.50000(0)
P	0.32320(87)	0.64468(82)	0.25487(71)
O1	0.37052(60)	0.23868(55)	0.58512(46)
O2	0.10648(72)	-0.33062(68)	0.36174(49)
O3	0.69842(71)	0.65649(64)	-0.13517(56)
O4	0.27216(78)	0.79624(73)	0.09621(49)
F	-0.11003(80)	0.08701(65)	0.24565(55)
Li	0.70790(270)	0.35916(358)	0.22114(234)

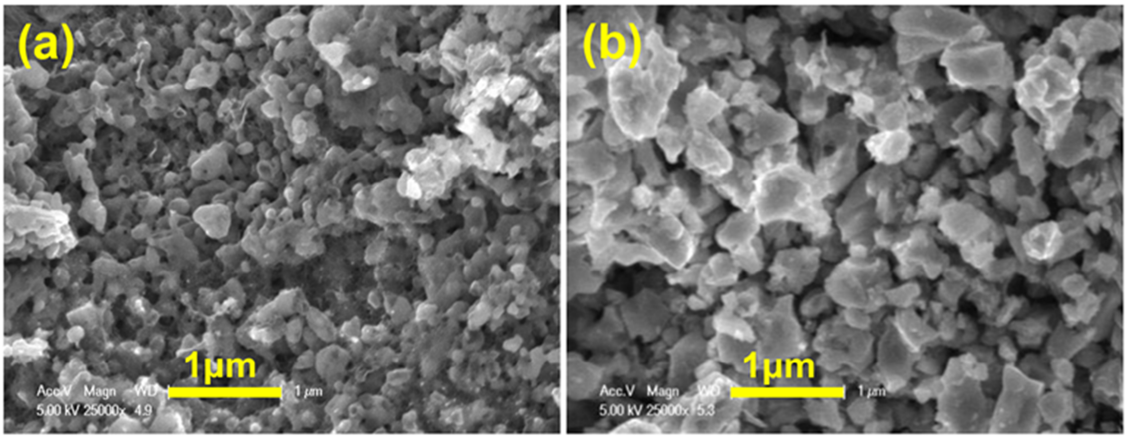

Figure S5. SEM images of the sample synthesized at different amount of stearic acid (a) carbon-coated $LiVPO_4F$ using stearic acid 5wt% (b) bare $LiVPO_4F$ without stearic acid. Both of them were synthesized by scalable single-step process with PTFE (25 wt%). After performing the carbon coating, particle size decreases from 200 - 600 nm (bare) to 50 - 200 nm.

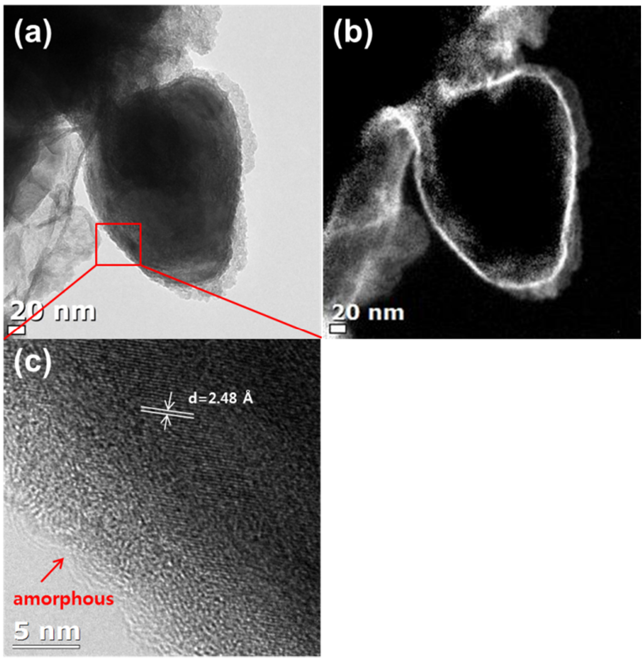

Figure S6. (a) TEM image of carbon-coated $LiVPO_4F$ synthesized by PTFE process (b) EELS mapping image of carbon layer. Carbon is well coated on the surface of submicron particles. (c) HR-TEM image. It shows amorphous carbon layer and lattice fringe of $LiVPO_4F$.

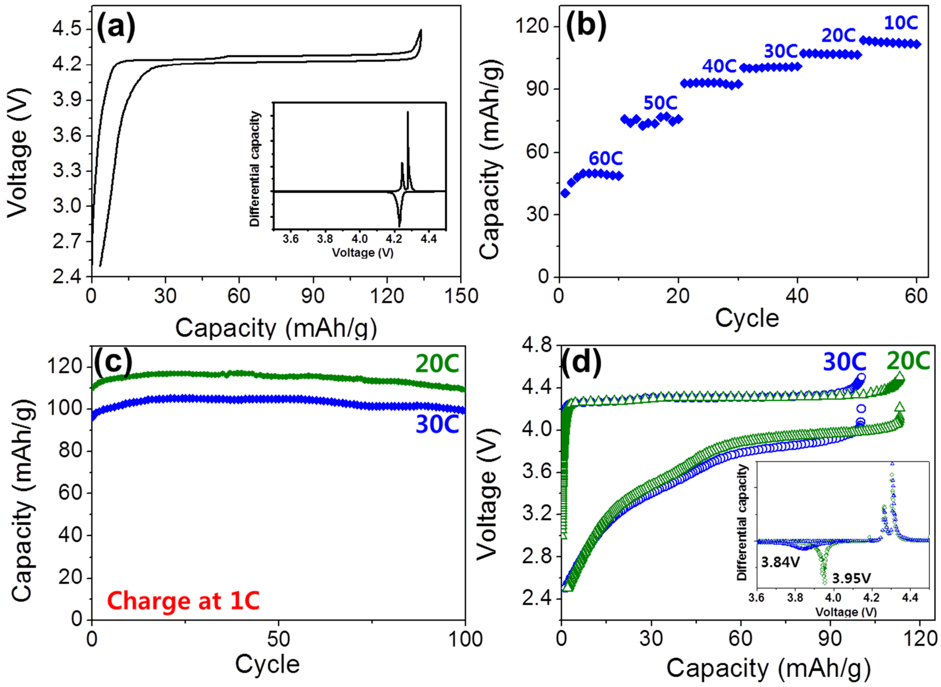

Figure S7. (a) Voltage profile of bare-$LiVPO_4F$ at C/10; (Inset: differential capacity (dQ/dV) plot of the voltage profile) (b) Rate capability at various discharge rates of bare-$LiVPO_4F$ (the cell was charged at 1C rate and discharged at various rates) (c) Capacity retention of bare-$LiVPO_4F$ at high discharge rates such as 20 C and 30 C rate when the cell was charged at 1C rate. (d) The voltage profiles of 2nd cycle in capacity retention test. Cut-off voltage was 2.5 V-4.5 V. (Inset: differential capacity (dQ/dV) plot of the voltage profiles at 20C and 30C rate).

Average working potentials at 20C and 30C discharge rate were higher than 3.45V, ~ 3.95 V for 20C and ~ 3.84 V at 30 C rate. For fabrication of the electrode, the ratio of the electrode was $LiVPO_4F$: carbon black (super P): binder (PVDF) = 80 :15 :5 (wt%). Loading density of electrode was ~ 2 mg/cm^2.

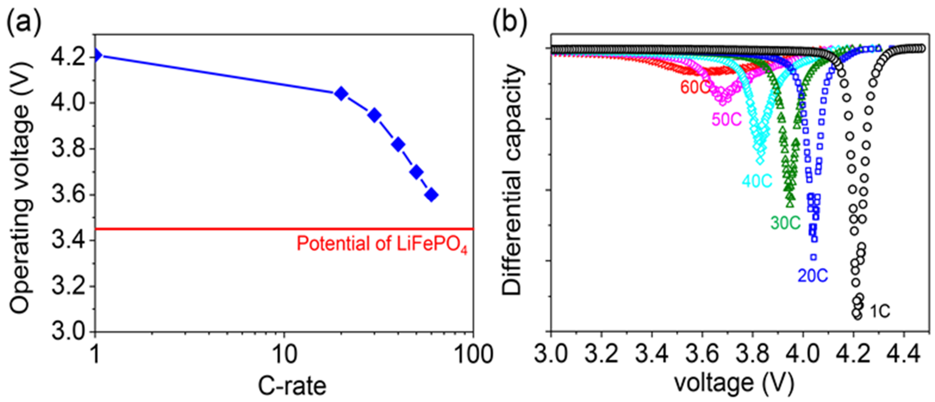

Figure S8. (a) Average operating voltages of carbon-coated $LiVPO_4F$ sample synthesized by PTFE process depending on different discharge rates. (b) Differential capacity (dQ/dV) plots of the discharge voltage profiles of carbon-coated $LiVPO_4F$ sample at various C-rates. The operating voltage (~ 3.6V) of carbon-coated $LiVPO_4F$ even at 60 C rate (1 min discharge) was much higher than 3.45V, the redox potential of $LiFePO_4$. This high operating potential with comparable capacity enables $LiVPO_4F$ to have higher energy density than $LiFePO_4$ at the same rate. (Voltage profiles from rate capability test in Fig. 6c)

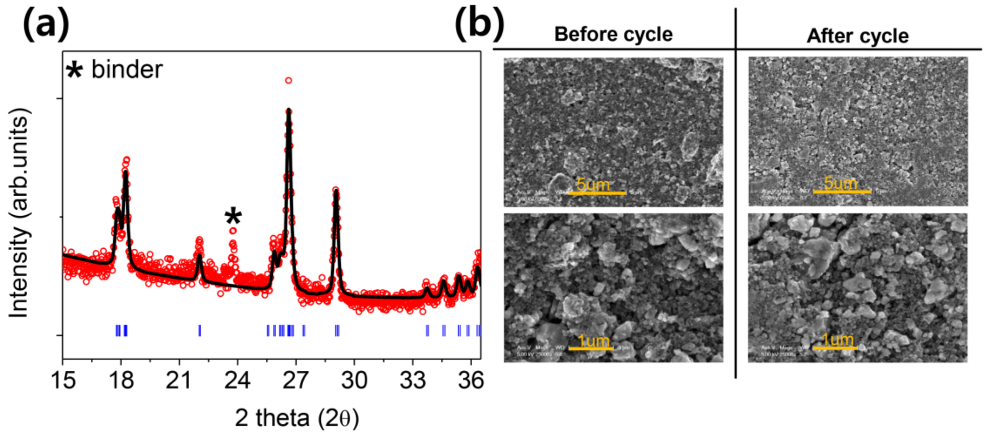

Figure S9. Cycle retention test of C-coated $LiVPO_4F$ at 10 C charge/10 C discharge. Other electrode was tested and it is confirmed stable cycle retention with similar capacity (a) XRD pattern and (b) SEM images of the electrode before and after 500 cycles.
